# Clinical Effects and Safety of Zhi Sou San for Cough: A Meta-Analysis of Randomized Trials

**DOI:** 10.1155/2017/9436352

**Published:** 2017-07-17

**Authors:** Ningchang Cheng, Jia Zhu, Pinpin Ding

**Affiliations:** ^1^Nanjing University of Chinese Medicine, Nanjing, Jiangsu 210023, China; ^2^Department of Respiratory Medicine, Jiangsu Province Hospital of Chinese Medicine, Affiliated Hospital of Nanjing University of Chinese Medicine, Nanjing, Jiangsu 210029, China

## Abstract

**Introduction:**

Zhi Sou San (ZSS), a traditional Chinese prescription, has been widely applied in treating cough. The purpose of this meta-analysis was to evaluate the effectiveness and safety of ZSS for cough.

**Methods:**

We searched relevant articles up to 5 March 2017 in seven electronic databases: the Cochrane Central Register of Controlled Trials (CENTRAL), MEDLINE, PubMed, Chinese National Knowledge Infrastructure (CNKI), Cqvip Database (VIP), China Biology Medicine disc (CBM), and Wanfang Data. Randomized controlled trials (RCTs) were eligible, regardless of blinding. The primary outcome was the total effective rate.

**Results:**

Forty-six RCTs with a total of 4007 participants were identified. Compared with western medicine, ZSS significantly improved the total effective rate (OR: 4.45; 95% CI: 3.62–5.47) and the pulmonary function in terms of FEV1 (OR: 0.35; 95% CI: 0.24–0.46) and decreased the adverse reactions (OR: 0.05; 95% CI: 0.02–0.01) and the recurrence rate (OR: 0.30; 95% CI: 0.16–0.57). However, there was no significant improvement in the cough symptom score comparing ZSS with western medicine.

**Conclusions:**

This meta-analysis shows that ZSS has significant additional benefits and relative safety in treating cough. However, more rigorously designed investigations and studies, with large sample sizes, are needed because of the methodological flaws and low quality of the included trials in this meta-analysis.

## 1. Introduction

Cough is the most common symptom among individuals seeking medical care. According to the duration, cough is divided into three types: acute, subacute, and chronic [1]. Acute cough (less than 3 weeks in duration) is the most predominant symptom of common cold or acute viral upper respiratory tract infection (URTI) [2, 3]. Acute cough from the common cold is usually transient and minor, but it may be life-threatening when it is caused by a serious illness [2]. Dyspnea, tachypnea, thoracic pain, hemoptysis, a severely worsened general state, and changes in vital signs are the major danger signs of acute cough [4]. Cough lasting from 3 to 8 weeks is categorized as subacute cough [5]. Postinfectious cough (PIC) is the most common cause of subacute cough [5]. Chronic cough is described as a cough that persisted for more than 8 weeks in adults [6] and more than 4 weeks in children (age < 15 years) [7]. Gastroesophageal reflux disease (GERD), asthma syndromes, smoker's cough, nonasthmatic eosinophilic bronchitis, and upper airway cough syndrome (UACS) associated with postnasal drip (rhinitis or rhinosinusitis) are the most common conditions associated with chronic cough in adults who are nonsmokers and are not receiving therapy with angiotensin converting enzyme (ACE) inhibitor [6, 8–10]. Unexplained chronic cough should be diagnosed as chronic cough with no etiology identified after evaluation and supervised therapeutic trial(s) that follow published best-practice guidelines [11].

Recent guidelines have attempted to provide directions in the treatment and management of cough. Patients with acute cough associated with the common cold can be treated with first-generation antihistamine/decongestant (A/D) preparation, expectorants, or mucolytics [3, 8]. Acute cough accompanying a cold or acute bronchitis/sinusitis usually resolves without any specific medicinal treatment. Any antibiotic treatment of uncomplicated upper respiratory tract infections should be avoided according to the guideline [4]. Although the inhaled ipratropium or inhaled corticosteroids (ICSs) may be useful for PIC, the optimal treatment is not known [8, 57]. Cough variant asthma (CVA) should be initially treated with inhaled bronchodilators and ICSs [8]. Dietary and lifestyle modification, acid suppression therapy, and prokinetic therapy are recommended in patients with chronic cough due to GERD [8]. Current recommendations on managing UACS include A/D or intranasal corticosteroids [6]. Multimodality speech pathology therapy and therapeutic trial of gabapentin are recommended for adults with unexplained chronic cough [11].

ZSS, a formula originating from Qing Dynasty, is commonly used in treating cough nowadays. Tan et al. [58], Zhang [59], and Meng et al. [60], respectively, reported that ZSS is the most frequently used formula in the treatment of PIC and CVA. ZSS is composed of seven herbs:* Platycodon grandiflorum*, Fine Leaf Schizonepeta herb, Tatarian Aster root, Sessile Stemona root/Japanese Stemona root/Tuber Stemona root, Willowleaf Swallowwort Rhizome, tangerine peel, and liquorice root. According to the theory of TCM, ZSS could loose the evil Qi and calm the lung Qi. Compared to its counterparts, ZSS is peaceful and gentle, not too cold or hot.

Modified Zhi Sou San (MZSS) could improve the symptoms of chronic obstructive pulmonary disease (COPD) in rats of northwest China with cold dryness syndrome and delay the velocity of decreased lung function [61]. An experiment showed that the antiasthmatic mechanisms of MZSS were related to its significant reduction in contents of endothelin-1 and nitric oxide, eosinophilia, and the damage of lung tissue [62].

Several systematic reviews and meta-analyses showed that ZSS might be effective in treating diseases-induced cough (including PIC, CVA, and laryngeal cough) [63–65]. Jing et al. reported that MZSS or MZSS combined with western medicine had better safety and efficacy than western medicine alone in treating PIC [63]. ZSS or ZSS combined with western medicine had superior effect and lower recurrence rate than western medicine alone in treating CVA [64]. Wang et al. reported that, compared with western medicine, ZSS had good efficacy and less adverse reactions in treating laryngeal cough [65]. Although the aforementioned reviews and meta-analyses elaborated that ZSS was more effective and safe than western medicine in treating diseases-induced cough, whether ZSS is the alternative medicine in treating cough is not confirmed. More trials and evidences are needed. So this meta-analysis was aimed at summarizing and evaluating the evidence from RCTs and determining whether ZSS is more effective and safer than western medicine in the treatment of cough.

## 2. Methods

### 2.1. Research Protocol

This meta-analysis is reported in accordance with Preferred Reporting Items for Systematic Reviews and Meta-Analyses (PRISMA) statement.

### 2.2. Databases and Search Strategies

We searched relevant articles up to 5 March 2017 in seven electronic databases: CENTRAL, MEDLINE, and PubMed in English and CNKI, VIP, CBM, and Wanfang Data in Chinese. The search terms used for databases were as follows: (cough or cough^*∗*^) for cough AND (zhisousan or zhisou san or zhisou powder) for ZSS AND randomized or controlled or clinical research.

### 2.3. Eligibility Criteria

Studies included had to meet the following criteria: (a) types of studies: any RCTs with ZSS or MZSS administrated orally in patients with cough were eligible, regardless of blinding; (b) types of participants: any patients diagnosed with cough regardless of sex, age, country, or underlying disease were included; (c) types of interventions: any variants of ZSS regardless of the herbs in the ZSS archetype replaced, added, or removed were included; the control group was taking the western medicine; (d) types of outcomes: the primary outcome was the total effective rate (clinical cure rate plus obvious cure rate plus showing effective rate). The clinical efficacy classified as clinical cure, obvious cure, and showing effective and not effective rate was based on the guiding principle of clinical research on new drugs of TCM and/or the diagnostic criteria of TCM syndrome. The secondary outcomes were the score of TCM symptom, cough symptom score (such as cough diary or visual analog scale), the adverse reactions, the pulmonary function test results, and the recurrence rate.

### 2.4. Study Selection and Data Extraction

Two independent investigators (Ningchang Cheng and Jia Zhu) screened the titles and abstracts of the searched articles. The trials obviously not meeting the inclusion criteria were excluded. We emailed the corresponding authors of the trials which possibly meet the inclusion criteria to ensure that the included trials were RCTs. Any disagreements were dissolved by consensus and discussions. All articles included were judged by the third reviewer (Pinpin Ding). Data extracted included the authors, year of publication, country, sample size, participants (mean age, cough duration), cough inducing disease, details of ZSS interventions, details of control inventions, treatment duration, outcome measurements, and adverse events [66].

### 2.5. Assessment of Risk of Bias

According to the Cochrane Handbook for Systematic Reviews of Interventions (version 5.0.2), the risk of bias was assessed in seven domains, such as random sequence generation and allocation concealment for selection bias, blinding of participants and personnel for performance bias, blinding of outcome assessment for detection bias, incomplete outcome data for attrition bias, selective outcome reporting for reporting bias, and other sources of bias.

### 2.6. Data Analysis

Review Manager Software (Version 5.3, Copenhagen, the Nordic Cochrane Centre, the Cochrane Collaboration, 2014) was used for data analysis. Heterogeneity between similar studies is evaluated by chi-square test and *I*^2^ statistic. There is moderate heterogeneity between studies, if *P* < 0.05 and *I*^2^ > 50%, and sensitivity analysis is needed. The enumeration data is evaluated as dichotomous data and expressed as odds ratio (OR) with 95% confidence interval (CI). The measurement data is evaluated as continuous data and expressed as mean difference (MD) with 95% CI. Statistical significant difference was considered as *P* < 0.05.

## 3. Results

### 3.1. Characteristics of the Included Studies

**Figure 1:**
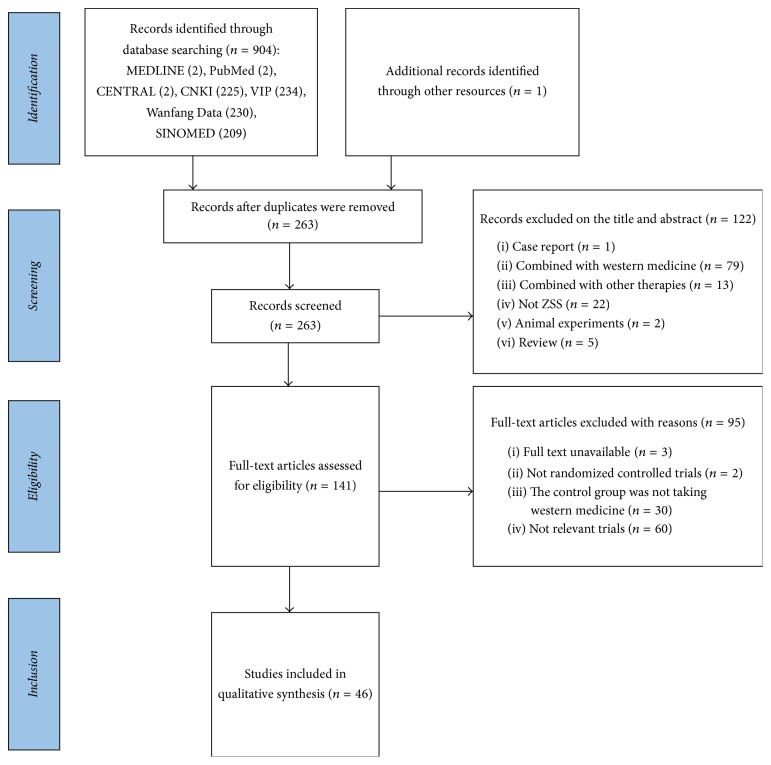
Flowchart of literature searching.

We identified 905 articles through electronic searching. After duplicates were removed, 263 records were screened. The full texts of 141 studies were assessed for eligibility after screening the titles and abstracts. 95 studies were excluded with reasons of full text being unavailable, not being RCTs, the controlled group not taking western medicine, and not relevant trials. Finally, 46 studies [12–56, 67] were included in this meta-analysis. A flowchart in the form of PRISMA is presented in Figure 1.

All the included trials originated from China and were published from 2004 to 2016. The total number of participants analyzed in the meta-analysis was 4007, of which 2077 received ZSS or MZSS, while 1930 received western medicine alone. The baseline characteristics of the included trials were shown in Table 1.

In the final selected studies, postsurgical cough accounted for one study [12], cough accounted for one study [43], allergic cough accounted for two studies [53, 54], acute cough accounted for one study [56], laryngeal cough accounted for six studies [31–35, 55], CVA accounted for eight studies [36–42, 67], chronic cough accounted for nine studies [44–52], and PIC accounted for eighteen studies [13–30].

### 3.2. The Total Effective Rate

**Figure 2:**
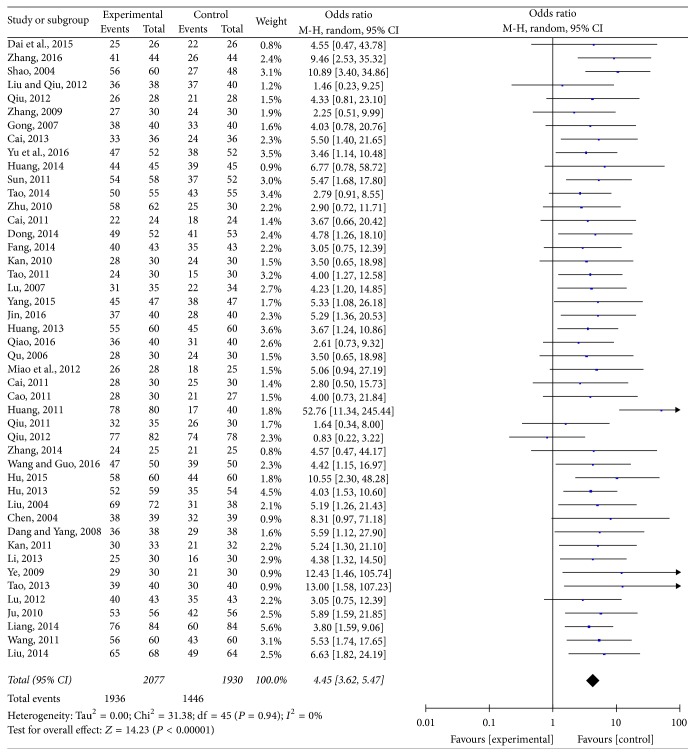
The effective rate comparing ZSS to western medicine alone.

All trials finally selected reported data on total clinical response rate [12–56, 67]. As for the fact that there was no significant heterogeneity (*I*^2^ = 0%; *P* = 0.94), a random-effects model was applied (Figure 2). The meta-analysis showed that a significant improvement in the total effective rate (OR: 4.45; 95% CI: 3.62–5.47) was observed when comparing ZSS to western medicine alone.

### 3.3. Cough Symptom Score

Five trials reported data on cough symptom score [17, 29, 41, 42, 67]. After sensitivity analysis, a trial [17] was excluded. As for the fact that there was no significant heterogeneity (*I*^2^ = 0%; *P* = 0.53), the meta-analysis showed that there was no significant improvement in the cough symptom score (OR: −0.72; 95% CI: −0.79–−0.65).

### 3.4. The Adverse Reactions

**Figure 3:**
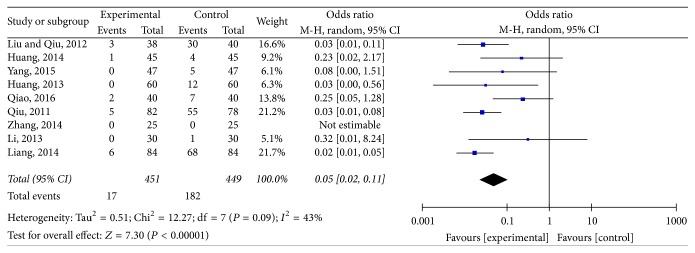
The adverse reactions comparing ZSS to western medicine alone.

Nine trials mentioned the adverse reactions as the secondary outcome [20–22, 26, 34, 46, 47, 50, 54]. As is shown in Figure 3, a random-effects model was applied; there was no significant heterogeneity (*I*^2^ = 43%; *P* = 0.09). The meta-analysis showed that a significant decrease in the adverse reactions (OR: 0.05; 95% CI: 0.02–0.01) was observed when comparing ZSS to western medicine alone.

### 3.5. The Pulmonary Function Test Results

**Figure 4:**
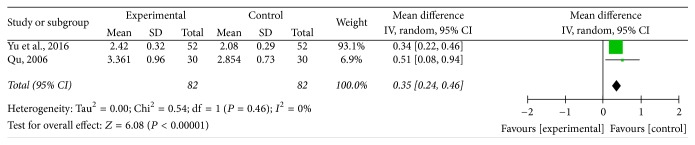
The pulmonary function test results comparing ZSS to western medicine alone.

Three trials provided data on the pulmonary function test results [36, 38, 41]. Two trials reported FEV1 (L) [38, 41]. The meta-analysis with random-effects model showed that ZSS significantly improved the pulmonary function in terms of FEV1 (*I*^2^ = 0%; *P* = 0.46; OR: 0.35; 95% CI: 0.24–0.46) in comparison to western medicine alone (Figure 4).

### 3.6. The Recurrence Rate

**Figure 5:**
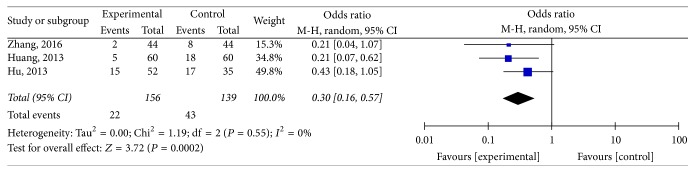
The recurrence rate comparing ZSS to western medicine alone.

There were three studies that talked about the recurrence rate [33, 34, 52]. Results (Figure 5) indicated that ZSS obviously decreased the recurrence rate compared with western medicine (*I*^2^ = 0%; *P* = 0.55; OR: 0.30; 95% CI: 0.16–0.57).

### 3.7. Assessing the Risk of Bias of the Included Studies

**Figure 6:**
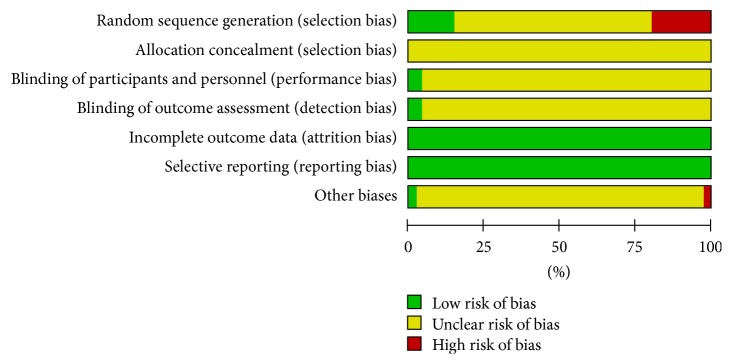
Risk of bias graph of the included trials.

**Figure 7:**
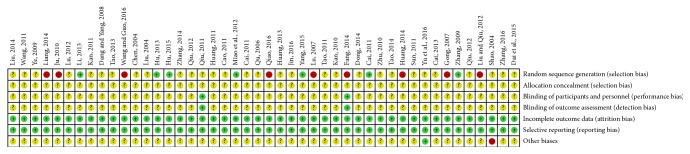
Risk of bias summary of the included trials.

The risk of bias of the finally included trials was not low. The selection bias was high due to the fact that wrong methods were applied in random sequence generation. Because multiple studies failed in blinding of participant and outcome assessment, the performance and detection biases were high (Figures 6 and 7) (+ indicates low risk of bias, − indicates high risk of bias, and ? indicates unclear risk of bias).

## 4. Discussion

A wide variety of pharmacological agents have been used in treating cough, such as antibiotic, ketotifen, asmeton, dextromethorphan, and ICSs [68]. No matter what caused cough, those mentioned agents were broadly used in remedying cough. “Zhisou,” by its Chinese definition, means relieving cough. So ZSS is one formula that has the behavior of relieving cough and it is widely applied in China, Korea, and Japan. It is necessary and attractive to compare ZSS and western medicine commonly used in curing cough. This meta-analysis was aimed at evaluating the effect and safety between ZSS and aforementioned western medicines. However, we could not find any studies originating from Japan or Korea in those databases that we have searched. It may be due to the fact that the authors of this meta-analysis all come from China; they do not master Japanese language and Korean language.

As we know, this meta-analysis is the first one about using ZSS in treating cough. This meta-analysis included studies of a wide range of conditions, such as acute cough, chronic cough, PIC, and CVA. The main findings of this meta-analysis are as follows: ZSS significantly improved the total effective rate and the pulmonary function in terms of FEV1, ZSS decreased the adverse reactions and the recurrence rate compared with western medicine, and ZSS appeared to be safe, well-tolerant, and more effective in treating cough.

However, we should admit that several limitations exist concerning this study. Firstly and foremost, the sample sizes of RCTs were small and limited. So, it was difficult to find out the influence of contingency factors. Secondly, insufficient reporting of random sequence generation and allocation concealment were the major methodological flaws in most of the included trials, which could result in selection bias and decrease the reliability of the evidence. Thirdly, the overall methodological quality of included trials was low due to the lack of blinding of participants and personnel and outcome assessment. On the whole, the finally included studies failed to follow CONSORT guidelines for RCTs; the risk of bias assessment was assessed as high risk or unclear risk in a majority of the RCTs. Therefore, RCTs with high quality and large sample are required to be done in the future.

## Figures and Tables

**Table 1:** Baseline characteristics of the included trials.

Author	Year	Country	Cough inducing disease	Sample size (*I*/*C*)	Intervention group	Control group	Age (years) (*I*/*C*)	Cough duration (*I*/*C*)	Intervention duration	Outcome measurements
Dong [12]	2014	China	Postsurgical cough	52/53	ZSS	Gentamicin + *α*-chymotrypsin	32–65	NR	3 d	①
Cai [13]	2013	China	PIC	36/36	MZSS	Ketotifen or ambroxol	6–15/7–19	NR	5 d	①
Cai [14]	2011	China	PIC	30/30	MZSS	Meptin + ketotifen + azithromycin	18–60/21–55	21–55 d/22–54 d	7 d	①
Cao [15]	2011	China	PIC	30/27	ZSS	Cephalexin + compound guaiacol potassium oral solution	3–8/4–10	15 d–1 y/10 d–10 m	7 d	①
Jin [16]	2016	China	PIC	40/40	MZSS	Pentoxyverine + loratadine	50.08 ± 7.82/49.85 ± 7.85	24.53 ± 8.40 d/23.88 ± 7.90 d	10 d	①
Ju [17]	2010	China	PIC	56/56	MZSS	Chlorphenamine maleate	37.5*l* ± 9.47/40.18 ± 10.56	40.5 ± 11.5 d/41.2 ± 12.7 d	15 d	① + ②
Kan [18]	2010	China	PIC	30/30	MZSS	Dextromethorphan + chlorphenamine maleate	45.47 ± 8.36/44.59 ± 8.72	4.8 ± 1.2 wk/4.6 ± 1.5 wk	10 d	①
Kan [19]	2011	China	PIC	33/32	MZSS	Roxithromycin id	2–10/2–10	4 wk–3 m/4 wk–3 m	14 d	①
Li [20]	2013	China	PIC	30/30	MZSS	Asmeton	32.19 ± 5.86/33.35 ± 5.72	36.6 ± 6.9 d/33.2 ± 7.5 d	7 d	① + ③
Liang [21]	2014	China	PIC	84/84	MZSS	Pentoxyverine	32.1 ± 1.7/31.9 ± 2.4	14.3 ± 2.0 d/14.0 ± 1.9 d	NR	① + ③
Liu and Qiu [22]	2012	China	PIC	38/40	MZSS	Pentoxyverine + chlorphenamine maleate	19–53/18–55	3–8 wk	NR	① + ③
Liu [23]	2014	China	PIC	68/64	MZSS	Ketotifen + carbetapentane citrate	36.0 ± 6.8/38.0 ± 7.5	33.0 ± 7.5 d/35.0 ± 8.5 d	10 d	①
Lu [24]	2012	China	PIC	43/43	MZSS	Ketotifen + meptin + azithromycin	34.3 ± 1.4	38 ± 2.1 d	7 d	①
Qiu [25]	2012	China	PIC	28/28	MZSS	Chlorphenamine maleate + cartussin	22–55/20–60	19–49 d/23–46 d	15 d	①
Qiu [26]	2011	China	PIC	82/78	MZSS	Dextromethorphan + loratadine	33.5 ± 8.6/34.3 ± 8.8	18 ± 3.0 d/17.6 ± 2.0 d	7 d	① + ③
Qiu [27]	2012	China	PIC	35/30	MZSS	Compound pholcodine syrup + asmeton + azithromycin	14–65/15–65	NR	3 d	①
Sun [28]	2011	China	PIC	58/52	MZSS	Chlorpheniramine maleate + methyl bromide	19–57/20–58	14–40 d/16–41 d	7 d	①
Tao [29]	2011	China	PIC	30/30	MZSS	Pseudoephedrine hydrochloride sustained release capsules	40.2 ± 8.7/39.5 ± 7.5	40.5 ± 13 d/42.8 ± 10.0 d	7 d	① + ②
Zhu [30]	2010	China	PIC	62/30	MZSS	Azithromycin	2 m–13/5 m–13	2–4 d/1–5 d	3 wk	①
Chen [31]	2004	China	Laryngeal cough	39/39	MZSS	Roxithromycin id + Zhikelu	5–87/7–82	5 d–2 y/3 d–1.5 y	5 d	①
Fang [32]	2014	China	Laryngeal cough	43/43	MZSS	Setastine + carbetapentane citrate + new bromide	40/41	3–12 wk/3–12 wk	14 d	①
Hu [33]	2013	China	Laryngeal cough	59/54	MZSS	Budesonide	34–63/32–64	3–8 wk/3–7.3 wk	7 d	① + ⑤
Huang [34]	2013	China	Laryngeal cough	60/60	MZSS	Cefuroxime axetil + phenergan syrup	16–64/17–65	7 d–3 m/7 d–3 m	10 d	① + ③ + ⑤
Liu [35]	2004	China	Laryngeal cough	72/38	MZSS	Amoxicillin or cefradine + phenergan cough syrup	31.3 ± 2.1/30.1 ± 2.3	42 ± 2.3 d/43.3 ± 6.7 d	4 d	①
Gong [36]	2007	China	CVA	40/40	MZSS	Ketotifen + doxofylline + salbutamol aerosol	41.3 ± 10.3/38.5 ± 11.8	2–36 m/2–30 m	28 d	① + ④
Lu [37]	2007	China	CVA	35/34	MZSS	Terbutaline + cetirizine	9.6/9.2	1.3 m/1.4 m	14 d	①
Liu [35]	2012	China	CVA	28/25	MZSS	Shah Mette Lo fluticasone propionate	43.79 ± 12.58/42.16 ± 10.77	3.42 ± 2.17 m/3.55 ± 2.29 m	14 d	① + ②
Qu [38]	2006	China	CVA	30/30	MZSS	Terbutaline	38.2 ± 12.5/40.3 ± 13.2	7.5 ± 8.5 m/8.0 ± 7.0 m	30 d	① + ④
Tao [39]	2014	China	CVA	55/55	MZSS	Montelukast sodium + budesonide aerosol	2–11/2.5–12	3–33 m/4–36 m	4 wk	①
Wang [40]	2011	China	CVA	60/60	MZSS	Conventional treatment for cough	2–9/2.5–10	NR	10 d	①
Yu et al. [41]	2016	China	CVA	52/52	MZSS	Shah Mette Lo fluticasone powder	40.9 ± 6.8/41.5 ± 7.2	8.5 ± 2.2 m/8.2 ± 2.4 m	30 d	① + ② + ④
Zhang [42]	2009	China	CVA	30/30	MZSS	Ceftazidime + dexamethasone	2–66	NR	14 d	① + ②
Shao [43]	2004	China	Cough	60/48	MZSS	Cefradine	14–66/12–62	NR	14 d	①
Cai [44]	2011	China	Chronic cough	24/24	MZSS	Conventional treatment for cough	26–70/20–65	8–36 wk/10–42 wk	14 d	①
Dai et al. [45]	2015	China	Chronic cough	26/26	MZSS	Desloratadine + ambroxol	37.83 ± 10.43/36.40 ± 11.89	28.55 ± 26.71 wk/34.73 ± 24.17 wk	14 d	①
Huang [46]	2014	China	Chronic cough	45/45	MZSS	Chlorphenamine + aminophylline + ambroxol	24–75	8 wk–2 y	14 d	① + ③
Qiao [47]	2016	China	Chronic cough	40/40	MZSS	Cefuroxime axetil + ambroxol + dextromethorphan	54.6 ± 7.9	3.3 ± 1.6 y	1 m	① + ③
Tao [48]	2013	China	Chronic cough	40/40	MZSS	Azithromycin + ambroxol	20–76	NR	7 d	①
Wang and Guo [49]	2016	China	Chronic cough	50/50	MZSS	Chlorpheniramine maleate + salbutamol + methyl bromide	4.65 ± 1.94/5.46 ± 2.53	NR	7 d	①
Yang [50]	2015	China	Chronic cough	47/47	MZSS	Asmeton	41.6 ± 5.7	10.4 ± 1.9	7 d	① + ③
Ye [51]	2009	China	Chronic cough	30/30	MZSS	Asmeton	19–46	NR	7 d	①
Zhang [52]	2016	China	Chronic cough	44/44	MZSS	Ambroxol	50 ± 2.96/51 ± 2.63	NR	14 d	① + ⑤
Dang and Yang [53]	2008	China	Allergic cough	38/38	MZSS	Ketotifen + aminophylline	2–12/2–12	1–6.6 m/1–5.9 m	7 d	①
Zhang [54]	2014	China	Allergic cough	25/25	MZSS	Terbutaline	6.25 ± 3.05/6.05 ± 3.12	8.09 ± 15.45 m/7.86 ± 15.36 m	4 wk	① + ③
Hu [55]	2015	China	Acute cough	60/60	ZSS	Asmeton	37 ± 11/36 ± 10	18 ± 6 d/16 ± 7 d	14 d	①
Huang [56]	2011	China	Acute cough	80/40	MZSS	Amoxicillin	19–35/20–34	0.5–7 d/0.5–6.5 d	3 d	①

y, year; m, month; wk, week; d, day; NR: not reported. *Note*. ① The total effective rate, ② cough symptom score, ③ the adverse reactions, ④ the pulmonary function test results, and ⑤ the recurrence rate.
